# Impact of Glucagon-Like Peptide-1 Receptor Agonists on Mental Health: A Systematic Review of Psychiatric Safety and Antidepressant Potential

**DOI:** 10.7759/cureus.115703

**Published:** 2026-09-03

**Authors:** Aisha Quadir, Roshna Devi, Bader Jad Allah, Abbas Haider, Sabah Shakeel Shaikh, Kalyanram N Botsa

**Affiliations:** 1 Internal Medicine/Family Medicine, California Institute of Behavioral Neurosciences and Psychology, Fairfield, USA; 2 Internal Medicine, California Institute of Behavioral Neurosciences and Psychology, Fairfield, USA; 3 Family Medicine, Seha Clinics, Abu Dhabi, ARE; 4 Ophthalmology, California Institute of Behavioral Neurosciences and Psychology, Fairfield, USA; 5 Diabetes and Endocrinology, California Institute of Behavioral Neurosciences and Psychology, Fairfield, USA; 6 Internal Medicine, Sri Ramachandra Institute of Higher Education and Research, Chennai, IND

**Keywords:** depression, glucagon-like peptide-1 receptor agonists, mental health, psychiatric safety, semaglutide, suicidality, systematic review

## Abstract

Glucagon-like peptide-1 receptor agonists (GLP-1RAs) are increasingly used for type 2 diabetes mellitus (T2DM) and obesity, but concerns about their mental health effects have been raised. Early safety reports linked them to suicidal thoughts and worsening depression; however, evidence remained mixed and unclear. A systematic search was conducted across three databases for studies published between 2021 and May 2026. Out of 617 records identified, 11 studies were finally included after quality checks using appropriate appraisal tools based on study design. This review was not prospectively registered.

Data from over 4.9 million patients across eleven primary studies showed that GLP-1RAs did not increase psychiatric risk overall. Ten out of eleven primary studies confirmed either safety or protective psychiatric effects. Increased psychiatric risk was seen in one obesity-specific study; this may partly reflect surveillance or detection bias, though this was not directly tested. Only one of the 11 included studies directly measured mood using validated questionnaires and found that semaglutide was associated with lower (better) depression and anxiety scores. GLP-1 receptor agonists appear to maintain a safe neuropsychiatric profile, with an emerging signal that mood may benefit, currently limited to a single scale-based study among those formally included; most existing evidence remains diagnostic-code-based rather than scale-based. For T2DM patients with stable mood disorders, there is no justification for withholding these medications. For weight management, clinicians may consider baseline mental health screening before starting treatment. Future research must use validated continuous mood rating tools rather than administrative diagnostic codes to properly measure psychological benefits.

## Introduction and background

Among patients with obesity and type 2 diabetes mellitus (T2DM), major depressive disorder (MDD) is one of the most common psychiatric comorbidities [1,2]. These conditions are closely linked through shared mechanisms such as chronic systemic inflammation, disrupted stress-hormone regulation via hypothalamic-pituitary-adrenal axis dysfunction, and overlapping metabolic factors. Clinicians are now more often prescribing glucagon-like peptide-1 receptor agonists (GLP-1RAs) such as semaglutide and liraglutide to treat obesity and diabetes [3,4]. GLP-1 is a naturally occurring gut hormone that regulates blood glucose and appetite. These drugs are incretin mimetics that work by mimicking its effects to improve glycemic control. They also promote weight loss by delaying gastric emptying and altering hypothalamic and limbic signaling, the brain circuits that govern emotion and reward [4-6]. They are now widely used for reducing cardiovascular risk and body weight [3]. However, their growing popularity has raised important questions about their effects on mental health and psychiatric outcomes.

GLP-1 receptors are expressed in brain regions that control both metabolic and mood states [6]. Researchers therefore want to know whether these agents have neuroprotective effects or directly alter the underlying biology that drives depression. Recent data suggest that they may help keep patients with concurrent metabolic and mental illness psychiatrically stable [2,7]. Even patients on antidepressant medications that cause weight gain appear to remain stable on GLP-1RAs [1].

Initially, GLP-1RAs raised notable safety concerns. Potential risks of suicidal ideation and acute or worsening depressive episodes were flagged in early post-marketing reports, safety data collected after a drug reaches the public, and safety registries [8,9]. After major studies across multiple countries, the U.S. Food and Drug Administration investigated and concluded that there is no link between GLP-1RAs and suicidal behavior [10]. Even so, a gap remains. Large studies relied on insurance billing records and diagnostic codes, which capture whether a diagnosis was made but not day-to-day mood changes. Small clinical studies used validated psychometric scales that captured these changes directly but included too few patients to draw firm conclusions. As a result, there is still no clear consensus on whether these medications prevent psychiatric hospitalization due to self-harm or severe depression.

Prior reviews have generally focused on psychiatric safety alone. This review also looks at antidepressant potential and gives more weight to scale-based evidence over diagnostic codes where both exist. This review aimed to address this gap by examining all available datasets. It specifically investigated the following. First, what is the current evidence regarding the psychiatric safety of GLP-1 receptor agonists? Second, to what extent do they demonstrate potential to mitigate depressive symptoms and lower the risk of psychiatric or mental health-related hospitalization secondary to depressive disorders or self-harm events? Clinical trials were compared with large registries to help guide safer medication choices for patients with mental illness.

Resolving this question carries direct clinical weight, since unwarranted caution could lead clinicians to withhold an effective treatment, while overlooking the signal could mean missing a meaningful mood benefit for patients who need it.

## Review

Materials and methods

This systematic review followed the Preferred Reporting Items for Systematic Reviews and Meta-Analyses (PRISMA) statement guidelines [11]. An initial search was performed that captured studies published from 2021 to May 2026; exact per-database search execution dates were not separately logged. Three primary databases were used: PubMed/MEDLINE, Google Scholar, and ScienceDirect. PubMed Central (PMC) was also searched, but its records were grouped with PubMed/MEDLINE rather than counted as a separate database, since PMC is a full-text archive, not an independent bibliographic database. The initial search gave a total of 617 records: 352 from PubMed/MEDLINE (including PMC), 125 from Google Scholar (out of approximately 149 total results, sorted by relevance, within the 2021-2026 date range), and 140 from ScienceDirect. This review was not registered with PROSPERO or another systematic review registry, which is acknowledged as one of the limitations.

*Search Strategy* 

Medical Subject Headings (MeSH) terms and Boolean operators (AND, OR) were used in PubMed/MEDLINE. Search terms were grouped into three conceptual domains: glucagon-like peptide-1 receptor agonists (e.g., "Glucagon-Like Peptide-1 Receptor Agonists"[Mesh], "Semaglutide"[Mesh], "Liraglutide"[Mesh]), mental health outcomes (e.g., "Depressive Disorder"[Mesh], "Mental Disorders"[Mesh], "Suicidal Ideation"[Mesh], "Psychiatric Status Rating Scales"[Mesh]), and hospitalization metrics (e.g., "Hospitalization"[Mesh], "Patient Admission"[Mesh], "adverse effects"[subheading]). 

The Google Scholar and ScienceDirect search terms shown in Table 1 represent the complete queries used for those databases. The 125 of approximately 149 Google Scholar results retained represent the first several pages of relevance-ranked output within the stated date range. Backward and forward citation chasing, trial registry searches, author contact, and grey literature searches were not performed, nor was citation or related article searching used. Searches were not conducted while logged into an institutional account. Table 1 shows the detailed search strategy along with the databases.

**Table 1:** Search strategy and databases searched * The third PubMed/MEDLINE search and the PubMed Central (PMC) search use the same query, run across two interfaces. Records are reported as retrieved; any overlap between them was resolved at the duplicate-removal step.

Search strategy	Database used	Number of papers identified
GLP-1 receptor agonists AND depression AND mental health	PubMed/MEDLINE	72
(((semaglutide OR liraglutide OR dulaglutide OR tirzepatide OR "glucagon-like peptide-1 receptor agonists") AND (depression OR "depressive disorder" OR "mood disorder")) AND ("adverse events" OR "psychiatric adverse events" OR "psychiatric safety" OR hospitalization OR "mental health hospitalization" OR "depressive symptoms" OR "depression scale")) AND (("2021"[Date - Publication]: "3000"[Date - Publication]))	PubMed/MEDLINE	131
("Glucagon-Like Peptide-1 Receptor Agonists"[Mesh] OR "Semaglutide"[Mesh] OR "Liraglutide"[Mesh]) AND ("Depressive Disorder"[Mesh] OR "Mental Disorders"[Mesh] OR "Suicidal Ideation"[Mesh] OR "Psychiatric Status Rating Scales"[Mesh]) AND ("Hospitalization"[Mesh] OR "Patient Admission"[Mesh] OR "adverse effects"[subheading])	PubMed/MEDLINE	102
("Glucagon-Like Peptide-1 Receptor Agonists"[Mesh] OR "Semaglutide"[Mesh] OR "Liraglutide"[Mesh]) AND ("Depressive Disorder"[Mesh] OR "Mental Disorders"[Mesh] OR "Suicidal Ideation"[Mesh] OR "Psychiatric Status Rating Scales"[Mesh]) AND ("Hospitalization"[Mesh] OR "Patient Admission"[Mesh] OR "adverse effects"[subheading])	PubMed/MEDLINE (PMC)*	47
GLP-1 receptor agonist AND anti-depressants	Google Scholar	125
GLP-1 receptor agonist AND anti-depressants	ScienceDirect	140

Eligibility Criteria

Inclusion criteria*: *Studies were included that were peer-reviewed and published in English and focused on FDA-approved GLP-1 receptor agonists. Tirzepatide, though a dual GIP/GLP-1 receptor agonist, was treated as an eligible GLP-1RA. Studies also had to report at least one relevant outcome: therapeutic efficacy (changes in depression symptoms or rating scales), psychiatric safety (adverse events), or psychiatric or mental health-related hospitalizations. Eligible study designs were randomized controlled trials, observational cohort studies, and cross-sectional studies. Both diabetes and obesity populations were eligible, including studies with mixed or overlapping populations. Pre-existing psychiatric disorder was not required for inclusion. No dose, duration, or route restriction applied beyond FDA-approved use.

Exclusion criteria:* *Studies were excluded if they were grey literature or non-peer-reviewed studies; animal or in vitro studies; or involved pregnant or lactating women. Studies that failed to report specific psychiatric safety outcomes, mood scales, or hospitalization data were not part of this review. Also, hospitalizations primarily due to a major neurologic disorder (e.g., dementia, Parkinson’s disease) rather than psychiatric or depressive events were excluded. Studies where the primary outcome or hospitalization was related to substance use disorder (e.g., alcohol use disorder, opioid use disorder) rather than mood or depressive episodes were not included. 

Studies were also excluded from the final synthesis if psychiatric assessment was not a primary research question. This included papers that relied on spontaneous adverse event reporting databases without defined patient cohorts or follow-up, or weight loss trials that only measured psychiatric outcomes as an afterthought. Similarly, Badulescu et al., 2026, is cited in the introduction as background support but was excluded from formal appraisal [7]. Its primary outcome was cognitive function, with depression and suicidality assessed only as secondary, exploratory measures [7].

Screening and Study Selection

After the initial search, results were exported to EndNote (Clarivate, Philadelphia, PA, USA) and duplicate records were removed. Two reviewers (A.Q. and R.D.) independently screened titles and abstracts, then reviewed the remaining full texts against the predefined inclusion and exclusion criteria to determine eligibility. Disagreements at both stages were resolved by consensus. No automation or machine-learning tools were used, and screening was not blinded to study author, institution, or journal. Similarly, English-language and peer-review-status restrictions were applied during eligibility screening rather than as search-engine filters.

Data Extraction

The same two reviewers independently extracted key variables from the included studies, with discrepancies resolved by consensus. Extracted elements included primary author, year of publication, study design, population demographics, type and dose of GLP-1RA intervention, baseline psychiatric status, diagnostic or psychometric metrics utilized (e.g., continuous rating scales [12,13] versus ICD diagnostic codes [14,15]), and key raw findings regarding therapeutic mood responses or acute hospitalization risks.

In this review, psychiatric worsening means new or escalating symptoms serious enough to need clinical care or hospitalization. Psychiatric hospitalization means inpatient admission for a primary psychiatric diagnosis. Suicidal ideation or behavior covers ideation, self-harm, attempts, and death.

We did not statistically pool the data, given heterogeneity in populations, comparators, and outcome definitions across the included studies; instead, a narrative synthesis was done. We grouped studies by outcome type: psychiatric safety and suicidality, mood scale results, and psychiatric hospitalization. Where a study reported more than one estimate, the adjusted, active-comparator version was preferred. The three outcome groups were treated as co-equal rather than ranked, and studies contributing to more than one group were discussed within the relevant sections. Effect measures matched what each study reported: hazard ratios for time-to-event outcomes, an incidence rate ratio for the case-series design, and group means with standard deviations for the scale-based outcome. Conflicting findings were discussed individually by population and design rather than pooled.

Quality Appraisal

Specific quality appraisals were done based on the study design. Cohort studies were appraised using the Newcastle-Ottawa Scale (NOS) [16] across the domains of selection, comparability, and outcome to establish clear risk-of-bias categorizations. Randomized controlled trials (RCTs) were evaluated using the Cochrane Risk of Bias (RoB 2) tool [17], which focused on the randomization process, deviations from intended interventions, and missing outcome data. The Joanna Briggs Institute (JBI) tool [18] was used for a cross-sectional study. These standardized assessments provided a uniform basis for interpreting the reliability of the synthesized data.

Results

Search Results and Study Selection

A total of 617 records were identified from the initial search across three databases: 352 from PubMed/MEDLINE (including PMC), 125 from Google Scholar, and 140 from ScienceDirect. All citations were exported to EndNote (Clarivate, Philadelphia, PA, USA), and 75 duplicate records were identified and removed.

Screening of the remaining 542 records was done next by going through their titles and abstracts. This phase filtered out 361 papers that did not match the basic scope of our review, leaving 181 reports that were retrieved for a deeper full-text screening. Following the full-text screening, a formal eligibility check was performed. During this stage, the strict inclusion and exclusion criteria were applied to ensure the data closely aligned with the research question, which led to the exclusion of 163 articles due to ineligible study designs (n=94), lack of psychiatric tracking or ineligible outcomes (n=47), or insufficient data (n=22). 

This rigorous screening left 18 papers in the broader review catalog. After quality appraisal, seven of these were excluded on predefined design and scope grounds, such as spontaneous report data structure or secondary/exploratory psychiatric focus. Included studies were narrowed down to the 11 final primary studies, as shown in the PRISMA 2020 flow diagram (Figure 1).

**Figure 1:**
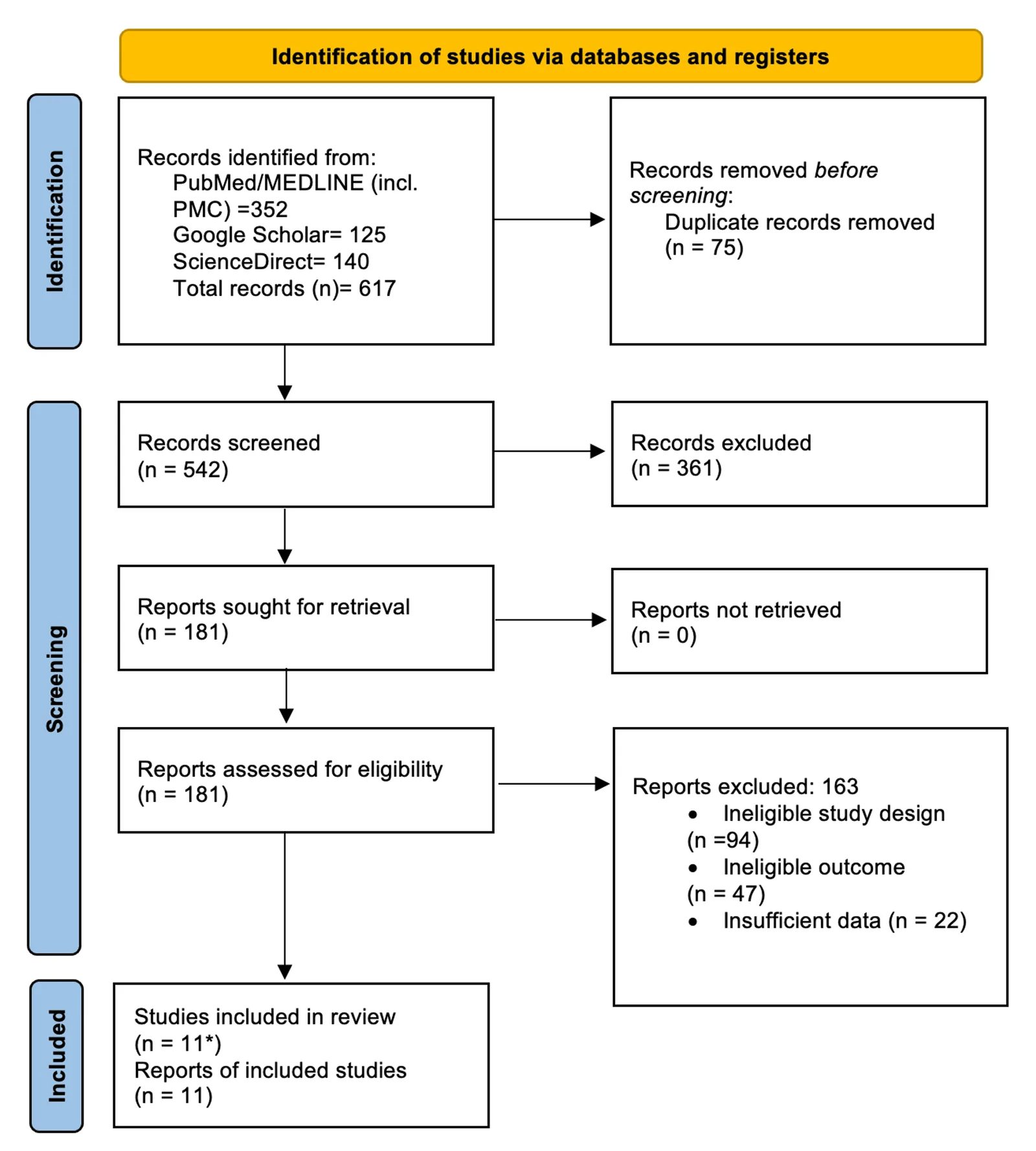
PRISMA 2020 flow diagram * Seven of the 18 eligible studies were excluded after quality appraisal: three RCTs (secondary psychiatric focus), one RCT (protocol only, no clinical outcomes), three pharmacovigilance studies (data structure incompatible with primary qualitative synthesis). PRISMA: Preferred Reporting Items for Systematic Reviews and Meta-Analyses; RCT: randomized controlled trial

Study Characteristics

Eighteen papers were grouped by their specific research designs. This helped determine the best quality appraisal tool for each type of study. The study designs and their corresponding assessment tools were categorized as follows.

Observational studies: This category represented the largest body of evidence. Ten were prospective or retrospective cohort designs (De Giorgi et al., 2024; Hurtado et al., 2024; Kornelius et al., 2024; Kuzucan et al., 2026; Shapiro et al., 2025; Stanislaus et al., 2026; Taipale et al., 2026; Tang et al., 2024; Ueda et al., 2024; Xie et al., 2025 [19-28]), and three were large-scale pharmacovigilance safety datasets (McIntyre et al., 2024; McIntyre et al., 2025; Battini et al., 2023 [8,9,29]).

Randomized controlled trials: Kushner et al., 2024; Wadden et al., 2024; Fang et al., 2026; Wadden et al., 2026 were the four RCTs [1,3-5].

Cross-sectional study: Witaszek et al., 2024, was the only cross-sectional study [30].

Supplementary evidence: An official U.S. Food and Drug Administration (FDA) 2026 regulatory statement was identified during screening but excluded from the primary synthesis, as it represents an institutional policy rather than primary research [10]. Kim et al., 2024, was identified after the original 18-paper set was finalized and, like the three pharmacovigilance datasets above, was not incorporated into the primary synthesis due to its data structure [31]. 

Table 2 presents the characteristics of the 18 studies assessed for quality.

**Table 2:** Study designs and quality appraisal tools of studies in review NOS: Newcastle-Ottawa Scale, RoB 2: Risk of Bias 2 tool, JBI: Joanna Briggs Institute [16-18]; RCT: randomized controlled trial

Paper	Study type	Assessment tool
De Giorgi et al., 2024 [19]	Cohort	NOS
Hurtado et al., 2024 [20]	Cohort	NOS
Kornelius et al., 2024 [21]	Cohort	NOS
Kuzucan et al., 2026 [22]	Cohort	NOS
Shapiro et al., 2025 [23]	Cohort	NOS
Stanislaus et al., 2026 [24]	Cohort	NOS
Taipale et al., 2026 [25]	Cohort	NOS
Tang et al., 2024 [26]	Cohort	NOS
Ueda et al., 2024 [27]	Cohort	NOS
Xie et al., 2025 [28]	Cohort	NOS
Battini et al., 2023 [29]	Observational/pharmacovigilance	NOS
McIntyre et al., 2024 [8]	Observational/pharmacovigilance	NOS
McIntyre et al., 2025 [9]	Observational/pharmacovigilance	NOS
Fang et al., 2026 [4]	RCT	Cochrane RoB 2
Kushner et al., 2024 [1]	RCT	Cochrane RoB 2
Wadden et al., 2024 [3]	RCT	Cochrane RoB 2
Wadden et al., 2026 [5]	RCT	Cochrane RoB 2
Witaszek et al., 2024 [30]	Cross-sectional	JBI

Risk of Bias in Included Studies

Thirteen observational studies were appraised using the Newcastle-Ottawa Scale; ten cohort studies were rated low risk and retained, while three pharmacovigilance datasets (McIntyre et al., 2024; McIntyre et al., 2025; and Battini et al., 2023 [8,9,29]) were rated moderate risk and excluded from primary synthesis due to their data structure. Four RCTs were appraised using Cochrane RoB 2 [17]; three, namely Kushner et al., 2024, Wadden et al., 2024, and Wadden et al., 2026 [1,3,5], were excluded because their psychiatric outcomes were secondary rather than primary, and Fang et al., 2026 [4] was excluded as a protocol-only study with no reported clinical outcomes. Full NOS and RoB 2 domain-level judgments are shown in Figures 2-4.

**Figure 2:**
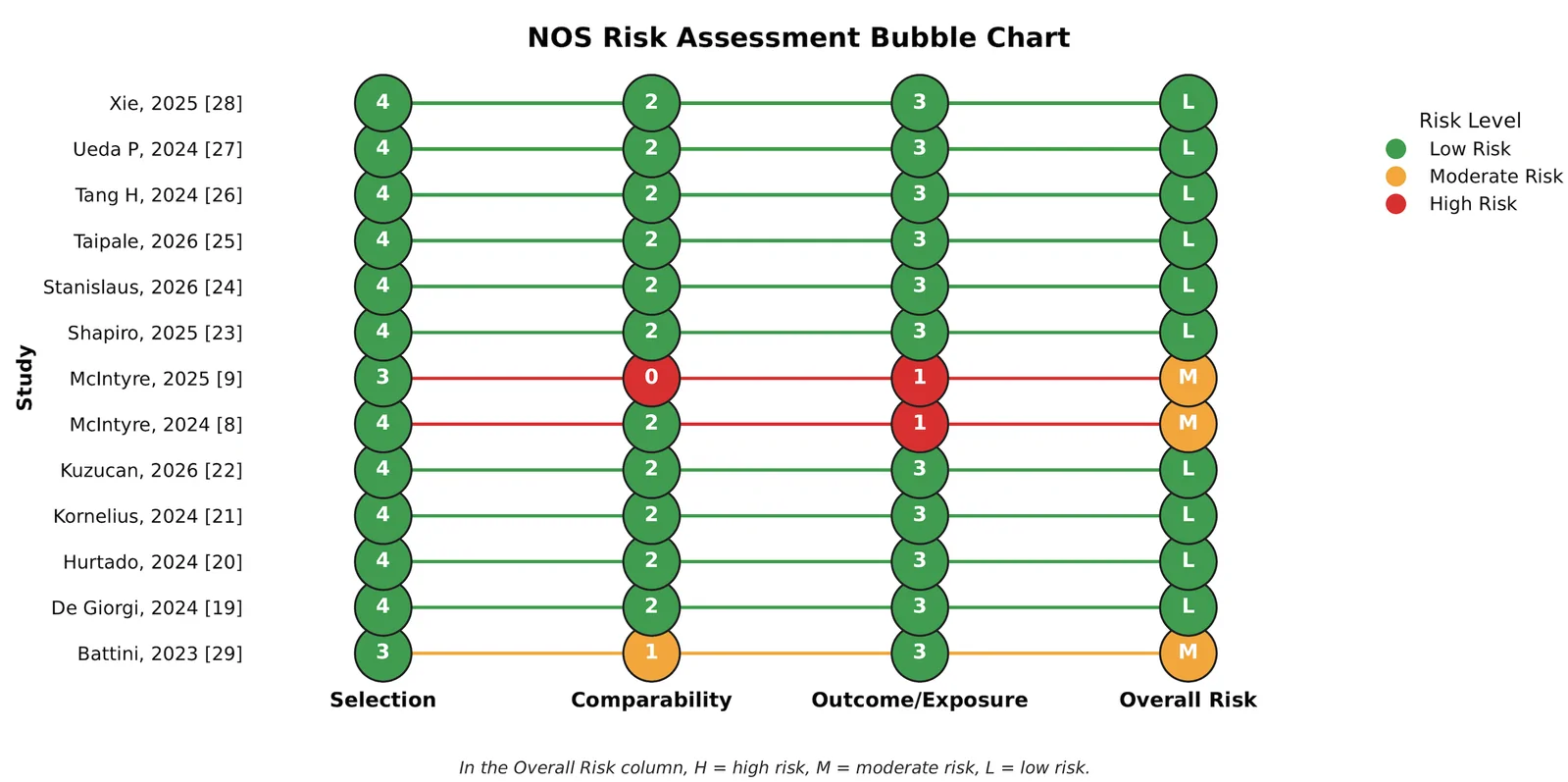
Newcastle-Ottawa Scale (NOS) risk-of-bias assessment of the observational studies

**Figure 3:**
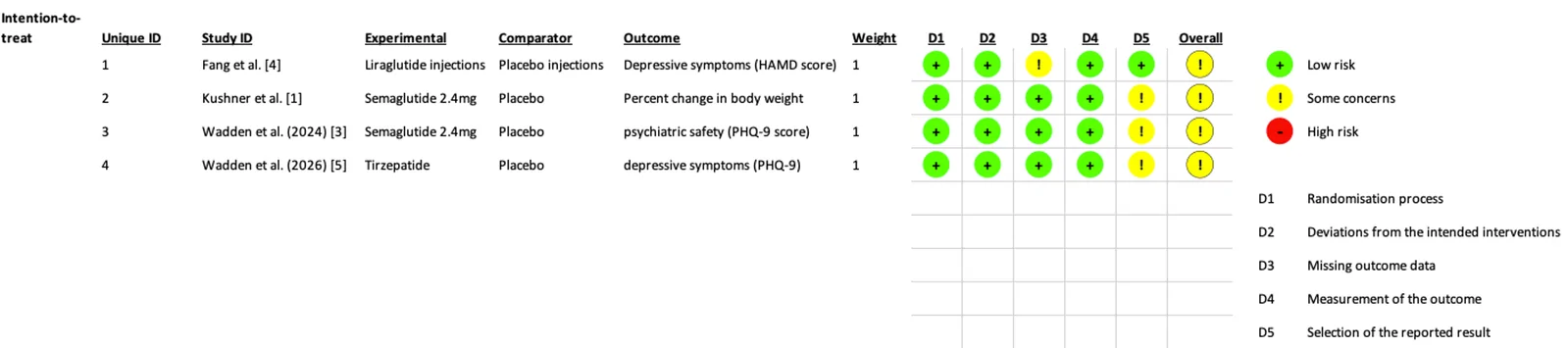
Cochrane Risk of Bias 2 (RoB 2) assessment of the four randomized controlled trials across the five domains

**Figure 4:**
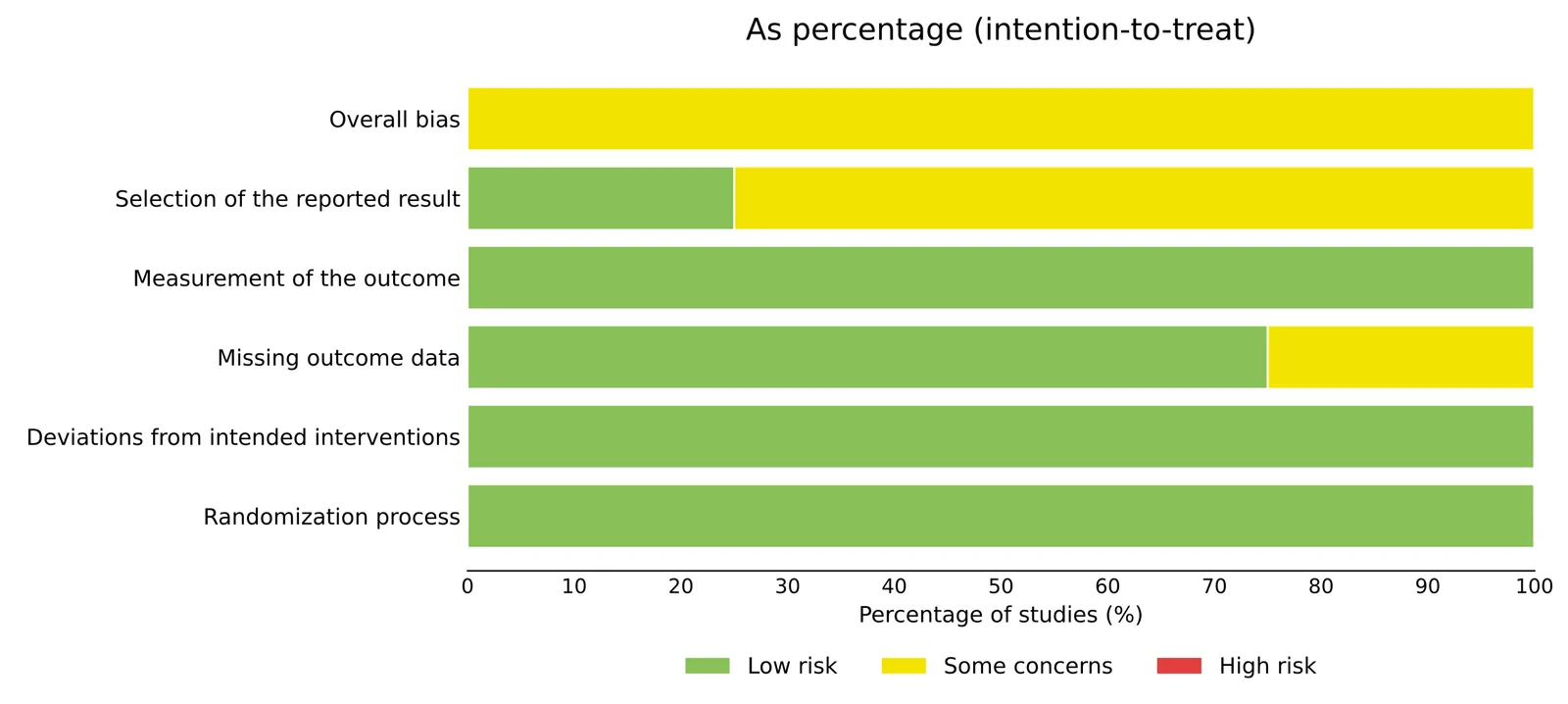
Risk-of-bias distribution across domains, shown as the percentage of randomized controlled trials at each risk level (intention-to-treat)

The single cross-sectional study, Witaszek et al., 2024 [30], met all JBI [18] criteria and was included, using validated psychometric instruments such as the PHQ-9 and GAD-7 [12,13]. As a result, the scale-based evidence in this review rests on this single cross-sectional study, a structural gap in the available literature rather than a limitation of this review's search strategy. The full JBI appraisal is shown in Table 3.

**Table 3:** Joanna Briggs Institute (JBI) appraisal of the cross-sectional study (Witaszek et al., 2024 [30])

JBI checklist criteria	Appraisal (yes/no)	Justification	Key findings
Inclusion criteria clearly defined?	Yes	The study clearly defines inclusion criteria for female Polish residents aged 18 and older, and specifies exclusion criteria for incompetent responses.	N/A
Study subjects and setting described in detail?	Yes	The study details the use of a computer-assisted web interviewing (CAWI) survey and recruitment through social media platforms.	N/A
Exposure measured in a valid and reliable way?	Yes	Exposure was measured using a standardised questionnaire to determine body mass index (BMI) categories and treatment history.	N/A
Standard/objective criteria for measurement of the condition?	Yes	The study employed internationally validated psychometric tools, specifically the PHQ-9 for depression and GAD-7 for anxiety.	N/A
Confounding factors identified?	Yes	Relevant confounding variables were identified, including age, BMI, chronic disease status, and history of bariatric surgery.	N/A
Strategies to deal with confounding factors stated?	Yes	The study used multivariable analysis and appropriate non-parametric statistical tests to account for identified confounding variables.	N/A
Outcomes measured in a valid and reliable way?	Yes	The primary outcomes regarding depression and anxiety symptoms were measured using valid and reliable standardised scales.	N/A
Statistical analysis appropriate?	Yes	The statistical approach included appropriate non-parametric tests such as Kruskal-Wallis, Mann-Whitney U, and Spearman's correlation.	Semaglutide treatment was associated with significantly lower Patient Health Questionnaire-9 (PHQ-9) scores (9.76 ± 6.37 vs. 10.84 ± 6.33) and Generalized Anxiety Disorder-7 (GAD-7) scores (8.71 ± 6.16 vs. 9.80 ± 6.07) compared to the rest of the study sample.

Participant and Study Characteristics

The 11 primary studies covered over 4.9 million patients from healthcare registries around the world [19-28,30]. All included patients were receiving treatment for either T2DM or obesity. No patients with primary dementia, advanced neurodegenerative conditions, or substance abuse were included.

The age range was wide, spanning younger adults, middle-aged people, and elderly patients (such as U.S. Medicare beneficiaries aged 66 or older) [26]. Sex distribution varied depending on the clinical condition being managed: (i) T2DM cohorts: These cohorts showed a roughly even sex distribution, with females making up 45% to 51% [22,27]; (ii) Obesity cohorts: Most patients were female, ranging from approximately 55% to 100% [21,30].

Synthesis of Clinical Findings

Overall, real-world observational data indicated that GLP-1RAs maintained a safe and potentially protective psychiatric profile.

Psychiatric safety and suicidality: Nine out of ten cohort studies found no elevated risk for neuropsychiatric events when GLP-1RAs were compared with other active antidiabetic medications [19,20,22-28]. Hazard ratios for suicidality or self-harm ranged from 0.93 to 1.07 in three of these studies, indicating minimal to no difference in risk compared with other antidiabetic medications [20,22,26].

Among these, two studies found a statistically significant protective effect. Multinational registry data reported an HR of 0.83 (95% CI 0.70-0.97), indicating a lower risk of the composite of suicide death and nonfatal self-harm [27]. Another large study of 215,970 GLP-1RA users found an HR of 0.90 (95% CI 0.86-0.94), which showed a statistically significant decrease in suicidal behaviors overall [28].

In contrast, one study, which looked at obese patients, found the opposite. It reported an approximately threefold increased risk of depression and a twofold increased risk of suicidal behavior [21]. This was a divergent finding, discussed further in the Discussion section.

Figure 5 presents the hazard ratios reported across the nine observational studies for psychiatric and suicidality outcomes [20-28]. As outcomes and comparators differed across studies, these are shown for descriptive comparison only and were not statistically pooled. Hazard ratios of eight studies were at or below 1.07 and directionally consistent with safety neutrality or a protective effect. Individual studies varied substantially in precision, however, with wide confidence intervals in some cases. Kornelius et al., 2024, reported a substantially elevated hazard ratio of 2.95, specifically for major depressive disorder, which stood as a divergent estimate in this comparison [21].

**Figure 5:**
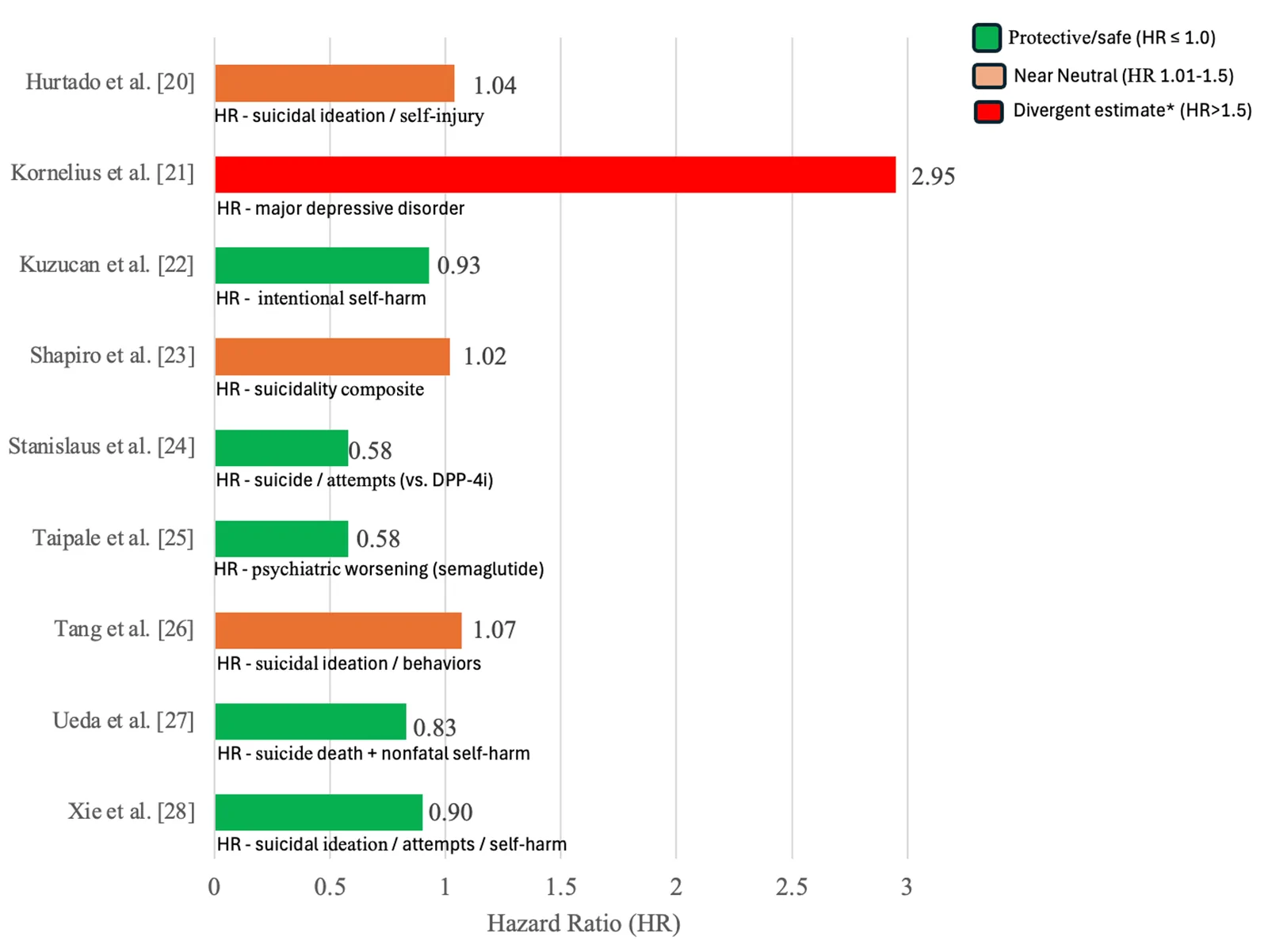
Hazard ratios for psychiatric and suicidality outcomes across nine included observational studies of GLP-1 receptor agonists Bars show point estimates only; confidence intervals for each study are reported in Table 4. HR ≤ 1.0 = protective/safe; HR 1.01-1.5 = near neutral; HR > 1.5 = divergent estimate. * Kornelius et al., 2024 - obesity-only cohort, no prior psychiatric history [21]. Elevated HR may partly reflect surveillance or detection bias (see Discussion). HR: hazard ratio; GLP-1: glucagon-like peptide-1

Antidepressant potential and mood scales: One out of eleven studies used validated mood scales to directly measure mood. In this specific study, GLP-1RA therapy showed a possible association with better mood. Semaglutide users (n=232) had significantly lower (better) depression scores (9.76 ± 6.37) and anxiety scores (8.71 ± 6.16) than the rest of the study sample (n=873; 10.84 ± 6.33 and 9.80 ± 6.07, respectively). Both results were statistically significant with p = 0.013 [30].

The other ten studies did not use mood questionnaires or scales; instead, they tracked whether patients received a new psychiatric diagnosis over time. One of these studies revealed that starting semaglutide reduced the risk of psychiatric worsening by 42% (aHR 0.58, 95% CI 0.51-0.65) and liraglutide reduced it by 18% (aHR 0.82, 95% CI 0.76-0.89) [25].

Risk of psychiatric hospitalization: There were only a few studies that could measure whether patients were admitted to an inpatient psychiatric hospital. The only available evidence came from one study that used national Swedish registry data [25]. When it counted only inpatient events, semaglutide lowered the risk of being hospitalized for a psychiatric reason or self-harm by 28% (aHR 0.72; 95% CI 0.59-0.89). The same study also found that GLP-1RAs as a class lowered self-harm risk by 44% (aHR 0.56; 95% CI 0.34-0.92). These two results overlap, since self-harm hospitalizations are already counted in the first number. 

The remaining studies could not measure this properly because they relied on administrative diagnostic codes, which usually cannot tell the difference between someone admitted to an inpatient psychiatric hospital and someone who just had an outpatient visit.

Table 4 summarizes the main characteristics of the 11 final studies included in this review.

**Table 4:** Summary of the 11 included primary studies GLP-1RA: glucagon-like peptide-1 receptor agonist(s); SGLT-2: sodium-glucose cotransporter-2 inhibitor(s); DPP-4: dipeptidyl peptidase-4 inhibitor(s); SCCS: self-controlled case series; SIS: suicidal ideation and self-injury; PHQ-9: Patient Health Questionnaire-9 [12]; GAD-7: Generalized Anxiety Disorder 7-Item Scale [13]; ICD-10/ICD-10-CM: International Classification of Diseases, 10th Revision/Clinical Modification [14]; ICD-9: International Classification of Diseases, 9th Revision [15]; OCD: obsessive-compulsive disorder; HR: hazard ratio; CI: confidence interval; ITT: intention-to-treat analysis; IRR: incidence rate ratio; aHR: adjusted hazard ratio

Study (author and year)	Study design	Population and follow-up	Intervention vs. comparator	Baseline psychiatric status	Outcome measure	Qualitative mood outcomes	Psychiatric safety
De Giorgi et al., 2024 [19]	Cohort study (observational)	Patients with type 2 diabetes mellitus; 65,176 patients total across three matched cohorts; 12-month follow-up.	Semaglutide vs. Sitagliptin, Empagliflozin, and Glipizide (active comparators).	Both incident (no prior diagnosis) and prevalent cases (with prior diagnosis) were analyzed.	ICD-10-CM diagnostic codes (clinical diagnosis).	No significant association found between semaglutide use and increased or decreased risk of depression, anxiety, or OCD compared to other antidiabetic agents. However, a reduced risk was observed for cognitive deficit and nicotine misuse.	No higher risk of psychiatric outcomes (including suicidality) observed; no statistically significant increase in risk was identified for semaglutide compared with other treatments.
Hurtado et al., 2024 [20]	Cohort study (observational)	Adult patients diagnosed with type 2 diabetes mellitus and concomitant obesity; 14,667 participants (3,040 initiating GLP-1RAs and 11,627 initiating SGLT-2is); cohort entry spanned from 2015 to 2021.	Glucagon-like peptide-1 receptor agonists (GLP-1RAs as a class; e.g., liraglutide and semaglutide mentioned contextually) vs. Sodium-glucose cotransporter 2 inhibitors.	Highly prevalent psychiatric comorbidities at baseline before adjustment. In the GLP-1RA group, 24.44% had depression, 49.44% had anxiety, 43.22% had sleep disorders, and 0.13% had a history of suicidal ideation and self-injury (SIS).	ICD-codes	Specific antidepressant efficacy or qualitative mood changes were not evaluated; the analysis concentrated purely on psychiatric adverse events.	No statistically significant difference in Suicidal Ideation and Self-injury (SIS) risk. Per-protocol HR: 1.04 (95% CI: 0.35, 3.14); Intention-to-treat HR: 1.36 (95% CI: 0.51, 3.61). Hospitalizations not isolated.
Kornelius et al., 2024 [21]	Cohort study (observational)	Adult patients (≥18 years old) diagnosed with obesity (BMI≥30); 324,506 (162,253 participants in the GLP-1RA cohort and 162,253 in the non-GLP-1RA cohort); Data window: 2015–2023. Individual follow-up tracked from 6 months up to 5 years.	GLP-1RAs, specifically Liraglutide and Semaglutide. vs. matched control individuals not using GLP-1RA medications.	Prior psychiatric history excluded to track new-onset risk (72,731 patients removed for baseline depression, anxiety, schizophrenia, bipolar, or suicidal behavior). Post-matching comorbidities included 5.2% psychoactive substance use.	ICD-10 mapped through the TriNetX platform database.	Not assessed; focus was strictly limited to diagnostic psychiatric adverse events.	Significantly increased risk found for major depressive disorder (HR 2.95; 95% CI: 2.82–3.08), anxiety (HR 2.08; 95% CI: 2.04–2.12), and suicidal ideation/attempts (HR 2.06; 95% CI: 1.92–2.21). Hospitalizations not isolated.
Kuzucan et al., 2026 [22]	Cohort study	Insured adult patients in the US diagnosed with type 2 diabetes; 3,639,520 total new users initially identified. Post-weighting comparison cohorts: 1,154,786 GLP-1RA users and 1,118,691 SGLT-2i users, 2015–2023. Average individual follow-up: 0.59 person-years (GLP-1RA) and 0.65 person-years (SGLT-2i).	GLP-1RAs as a class (including standard agents and the dual agonist tirzepatide) vs. SGLT-2 inhibitors (primary active comparator) and DPP-4 inhibitors (secondary active comparator).	High baseline psychiatric burden: 61.1% of the GLP-1RA cohort had a history of psychiatric illness, 50.1% had an active psychiatric diagnosis, and 0.1% had a history of intentional self-harm.	ICD-10-CM diagnostic codes.	Not assessed; focus was strictly limited to safety endpoints (suicidal ideation excluded due to claims data constraints).	No increased risk was identified. Adjusted hazard ratio for intentional self-harm showed no significant difference compared to SGLT-2is (HR 0.93; 95% CI: 0.81–1.08) or DPP-4is (HR 0.94; 95% CI: 0.82–1.07). Hospitalizations not isolated.
Shapiro et al., 2025 [23]	Cohort study	Adult patients in the United Kingdom diagnosed with type 2 diabetes; Two separate comparative cohorts: Cohort 1: N=270,110, Cohort 2: N=128,548; Median individual follow-up: 1.3 years vs. DPP-4i and 1.2 years vs. SGLT-2i.	GLP-1RAs as a class (including exenatide, liraglutide, lixisenatide, dulaglutide, and semaglutide). vs. DPP-4 inhibitors (Cohort 1) and SGLT-2 inhibitors (Cohort 2).	Patients with pre-existing mental health conditions were explicitly included. GLP-1RA groups carried a heavy baseline burden: ~46.5% depression, ~23-24% anxiety, and ~4.5% history of self-harm.	ICD-10 diagnostic codes via linked electronic healthcare databases.	Not assessed; focus was strictly limited to diagnostic clinical safety endpoints.	No increased risk was identified after adjustment. Weighted HR for the suicidality composite was 1.02 (95% CI: 0.85–1.23) vs. DPP-4is and 0.91 (95% CI: 0.73–1.12) vs. SGLT-2is. Self-harm isolated via hospital admissions.
Stanislaus et al., 2026 [24]	Cohort study paired with a matching Self-Controlled Case Series (SCCS) analysis.	Adult patients (≥18 years old) diagnosed with type 2 diabetes across the Danish national registry; Three specific allocations: • Cohort 1: N=161,830 (83,464 GLP-1RA vs. 78,366 SGLT-2i). • Cohort 2: N=163,733 (108,322 GLP-1RA vs. 55,411 DPP-4i). • SCCS Cohort: N=199,845 unique medication initiators. Median individual follow-up: 1 to 2 years (Intention-to-Treat) and 1 year average (As-Treated analysis).	GLP-1 receptor agonists vs. SGLT-2 inhibitors (primary active comparator) and DPP-4 inhibitors (secondary active comparator).	Evaluated via a 10-year lookback window. High psychiatric burden: 20% of the GLP-1RA cohort had a history of mental health conditions, including 13% antidepressant users and ~3% prior self-harm.	ICD-10 diagnostic mapping codes via the Danish National Patient Register.	Not assessed; focus was strictly limited to definitive clinical safety endpoints.	No increased risk was identified. Weighted ITT hazard ratios for suicide/self-harm showed neutral risk vs. SGLT-2is (HR 0.93; 95% CI: 0.57–1.52) and a protective decrease vs. DPP-4is (HR 0.58; 95% CI: 0.37–0.91). SCCS models mirrored the protective effect, IRR 0.45 (95% CI: 0.10-0.50). Hospitalization records isolated endpoints.
Taipale et al., 2026 [25]	Nationwide registry-based cohort study	Swedish adult patients diagnosed with depression, anxiety, or both, who initiated second-line non-insulin antidiabetic therapies; 95,490 total individuals, including 22,480 GLP-1RA users (13,445 semaglutide; 10,534 liraglutide; subgroups overlap because some patients used more than one GLP-1RA). Mean overall follow-up: 5.2 years. Median single continuous treatment use: 123 days for semaglutide and 61 days for liraglutide.	GLP-1RAs tracked as a class and individually (semaglutide, liraglutide, exenatide, dulaglutide). vs. Periods of non-use within the same individual (primary) and active head-to-head tracking against empagliflozin, dapagliflozin, or sitagliptin.	100% of the cohort had pre-existing psychiatric illness: 54.9% depression, 81.5% anxiety, and 36.4% diagnosed with both at entry.	ICD-10 administrative diagnostic entry codes (F32–F33, F40–F43) via health registries; no psychometric mood rating scales used.	Not assessed; focus was strictly limited to definitive clinical safety and diagnostic escalation endpoints.	Significant protective outcomes. Semaglutide (aHR 0.58; 95% CI: 0.51-0.65) and liraglutide (aHR 0.82; 95% CI: 0.76-0.89) significantly decreased worsening of mental illness. Class-wide GLP-1RA use reduced self-harm risk (aHR 0.56; 95% CI: 0.34–0.92). Semaglutide lowered inpatient psychiatric or self-harm hospitalizations (aHR 0.72; 95% CI 0.59-0.89).
Tang et al., 2024 [26]	Cohort study	U.S. Medicare beneficiaries (≥66 years old) diagnosed with type 2 diabetes; • Cohort 1: N=43,614 (21,807 matched pairs vs. SGLT2i). • Cohort 2: N=42,804 (21,402 matched pairs vs. DPP4i); Median individual follow-up tracking: 1.56 years (vs. SGLT2i cohort) and 1.64 years (vs. DPP4i cohort).	GLP-1 receptor agonists evaluated as a class and individually (liraglutide, dulaglutide, semaglutide, exenatide) vs. SGLT2 inhibitors (Cohort 1) and DPP4 inhibitors (Cohort 2).	Patients with a prior history of suicidal ideation/behavior were explicitly excluded. High background depression rates remained: 37.1% (SGLT2i cohort) and 40.9% (DPP4i cohort).	ICD-9 and ICD-10. No clinician-administered or patient self-report psychometric scales used.	Not assessed; investigative parameters were limited strictly to clinical administrative event codes.	No increased risk was identified. Composite ITT hazard ratios for suicidal ideation/behavior showed neutral risk vs. SGLT2is (HR 1.07; 95% CI: 0.80–1.45) and vs. DPP4is (HR 0.94; 95% CI: 0.71–1.24). Hospital/care settings embedded in diagnostic tracking.
Ueda et al., 2024 [27]	Cohort study	Adult patients (18 to 84 years old) treated in routine clinical practice across Sweden and Denmark; 298,553 total adults; Mean overall follow-up period: 2.5 years (2.7 years for GLP-1RAs; 2.3 years for SGLT2is).	GLP-1 receptor agonists as a class, primarily comprised of liraglutide (50%) and semaglutide (41%), vs. active comparison against sodium-glucose cotransporter-2 (SGLT2) inhibitors.	Broad population-based clinical registries. Subgroup analyses specifically tracked and included those with a recorded history of psychiatric disorders.	ICD diagnostic codes via national prescription and cause-of-death registers; no clinician-administered or patient self-report psychometric scales used.	Not assessed; clinical evaluations were restricted entirely to definitive diagnostic administrative events.	No increased risk was identified. Primary suicide death pooled risk was neutral (HR: 1.25; 95% CI: 0.83–1.88). Composite of suicide death and nonfatal self-harm showed an HR of 0.83 (95% CI: 0.70–0.97). Incident depression/anxiety showed an HR of 1.01 (95% CI: 0.97–1.06). Hospitalizations isolated via national patient registers.
Witaszek et al., 2024 [30]	Cross-sectional	Adult female patients in Poland across different weight categories undergoing anti-obesity interventions; 1105 adult women; Single-point cross-sectional data collection design; follow-up monitoring latency was omitted.	Semaglutide, liraglutide, and naltrexone/bupropion tracked within active anti-obesity clinical contexts vs. Comparative analysis across varying obesity tiers (Classes I, II, III), bariatric surgery status, and non-treated controls.	High background burden: 19.1% carried a formal diagnosis of depression, and 13.8% had an anxiety disorder. Active screening revealed 50.3% positive for depression and 46.4% positive for anxiety.	Standardized, validated psychometric continuous rating scales: PHQ-9 (depression severity; cut-off ≥12) and GAD-7 (anxiety screening).	Assessed and positive. Tracked as a continuous variable using quantitative point drops on psychological scales. Semaglutide patients demonstrated significantly lower (better) overall scores for both daily anxiety and depressive symptoms compared to the rest of the study population.	Significant qualitative psychological benefit observed. Semaglutide treatment was explicitly associated with significantly lower scores for anxiety (8.71±6.16 vs. 9.80±6.07, p=0.013) and lower scores for depression (9.76±6.37 vs. 10.84±6.33, p=0.013). Liraglutide did not meet statistical significance. Hospitalization administrative registries were omitted.
Xie et al., 2025 [28]	Cohort study	Adult patients diagnosed with type 2 diabetes initiating baseline anti-hyperglycemic therapies; 215,970 GLP-1RA users compared against active cohorts, including 258,614 SGLT2i users and 1,203,097 usual care controls. Data capture window: October 2017 to December 2023. Patient follow-up was dynamically censored upon treatment discontinuation or prescription refills delayed >90 days.	GLP-1 receptor agonists vs. Sulfonylureas, SGLT2 inhibitors, DPP4i and a usual care continuation group.	Broad population-based real-world clinical registry; baseline psychiatric variables were tracked via high-dimensional algorithmic covariates to balance baseline mental health history.	Administrative electronic health record billing entries; standard clinician-administered or patient self-report psychometric mood rating scales were not utilized.	Omitted; investigative parameters were restricted strictly to formal clinical event database logging.	Significant protective psychiatric outcomes observed. Class-wide GLP-1RA use significantly reduced the composite risk of suicidal ideation, suicide attempt, or intentional self-harm (HR: 0.90; 95% CI: 0.86–0.94).

Discussion

Interpretation of Principal Findings and Clinical Context

Across 11 primary studies covering over 4.9 million patients worldwide, the neuropsychiatric safety profile of GLP-1RAs appeared considerably more reassuring than regulatory concerns previously suggested. Pierret et al., 2025, recently published a meta-analysis of 80 placebo-controlled trials on this same question [32]. Our review takes a different approach, drawing on real-world observational and registry data instead of pooled trial results. This lets us capture patients whom trials often exclude, such as those with a pre-existing psychiatric history, and track outcomes trials rarely observe over their shorter follow-up, like psychiatric hospitalization. Nine out of ten cohort studies that were included in this review confirmed either safety neutrality or a protective psychiatric profile. The single cross-sectional study pointed to a mood benefit.

This is particularly important given the public anxiety that arose when early post-marketing surveillance flagged potential links between these medications and suicidal ideation and worsening depressive episodes. The U.S. Food and Drug Administration investigated this concern thoroughly and concluded that no causal link exists between GLP-1RA exposure and suicidal behaviors, which adds considerable weight to the safety profile [10].

Whether prescribed for glycemic control in T2DM or weight management, GLP-1RAs do not appear to trigger new-onset psychiatric illness or worsen clinical status in those with pre-existing mental health conditions. Instead, the literature collectively points toward a predominantly neutral safety profile with meaningful signals of an underlying protective influence. This protective quality becomes more visible in the reduction of severe, high-tier psychiatric decompensations.

The underlying pathophysiology of the protective quality of these agents aligns with current neurological models of GLP-1 signaling. GLP-1 receptors are widely expressed in central metabolic and emotional regulatory areas, including the hypothalamus, amygdala, and hippocampus [6]. They modulate neuroinflammatory pathways and dopaminergic transmission networks critical for emotional regulation, reward processing, and stress response.

Additionally, GLP-1RAs induce peripheral metabolic stabilization that provides substantial indirect psychological benefits by reducing chronic systemic inflammation, stabilizing glucose curves, and facilitating meaningful weight reduction. All of these remove significant physiological and psychological burdens that are known to sustain depressive pathophysiology.

Neuropsychiatric Safety Profile and Suicidality Paradox

Psychiatric safety evaluation and, more specifically, suicidal risk from the treatment have been the primary analytical focus of recent observational data. Kuzucan et al., 2026, evaluated a cohort of over 3.6 million new medication initiators managing T2DM using the U.S. FDA Sentinel system database [22]. This large-scale registry data confirmed that GLP-1RA exposure did not elevate the risk of intentional self-harm compared with active, real-world controls.

Beyond safety neutrality, the evidence also points toward potential risk reduction. Xie et al., 2025, compared GLP-1RAs with large standard-of-care control groups and identified a statistically significant 10% reduction in the composite risk of suicidal ideation, suicide attempts, and intentional self-harm [28]. This is not an isolated finding. Ueda et al., 2024, used routine clinical records from national databases across Sweden and Denmark and also reported no statistically significant increase in suicide death risk (HR 1.25, 95% CI 0.83-1.88), though the estimate was numerically increased and imprecise [27]. There was also a significant reduction in the composite of suicide death and non-fatal self-harm (HR 0.83, 95% CI 0.70-0.97) [27]. Wang et al., 2024, a large real-world cohort of over 1.8 million patients identified after our initial search, similarly found semaglutide use associated with a reduced risk of suicidal ideation [33]. The study by Stanislaus et al., 2026, further reinforced this protective signal [24]. They used a matched Self-Controlled Case Series design. It confirmed a sharp decrease in self-harm risk when GLP-1RA users were compared with patients taking DPP-4 inhibitors.

The observational results of the Kornelius et al., 2024, study remain a critical point of disclosure and public health anxiety [21]. The study reported a sharp increase in psychiatric risk (nearly threefold for major depressive disorder and roughly twofold for suicidal behavior), which stands as a divergent finding [21]. However, carefully dissecting this variance revealed vital epidemiological distinctions that contextualize the elevated risk.

First, the study by Kornelius et al., 2024, specifically isolated an anti-obesity population with no prior history of mental illness [21]. A total of 72,731 patients were excluded before matching for baseline depression, anxiety, schizophrenia, bipolar disorder, or suicidal behavior [21]. Thus, the elevated risk signals represent entirely new-onset psychiatric events occurring in previously psychiatrically healthy obese individuals. Every other study in this review predominantly tracked a different population of patients with pre-existing metabolic and psychiatric comorbidities.

Second, most importantly, patients seeking specialized weight-loss medications underwent considerably more frequent mental health screenings and intensive clinical interactions as compared with average primary care diabetes patients. This intensified clinical scrutiny may partly explain the higher rate of diagnostic code capture, though this was not directly tested. Hooker et al., 2026, a target-trial-emulation study identified after our initial search, similarly reported an increased incident-depression risk relative to two comparators [34]. This suggests surveillance bias alone may not fully account for the elevated risk seen in obesity-focused cohorts [34]. In a busy primary care diabetes clinic, it is far more likely that a psychiatric problem goes undetected than in a specialized obesity program, which can formally record psychiatric problems with structured mental health screening protocols.

In the study by Hurtado et al., 2024, patients managing T2DM often viewed their medication as a routine, physiological necessity [20]. In contrast, those individuals who are actively seeking weight-loss therapies may face markedly higher emotional stakes regarding body image and performance. It is possible that emotional distress following unmet weight-loss expectations contributes to some of these recorded diagnoses, though this was not directly measured in the included studies. These disparities contribute to the phenotypic variation in psychiatric outcomes observed between obesity and diabetes cohort studies. Future obesity-focused GLP-1RA studies should address this by mandating baseline PHQ-9 and GAD-7 screening for all participants [12,13]. This would let investigators distinguish genuine treatment-emergent psychiatric events from pre-existing conditions that simply went unnoticed before the more intensive clinical screening began.

Psychometric Granularity Versus Diagnostic Stability

A significant and pervasive limitation across the current literature is a distinct scarcity of direct psychometric testing. The majority of included studies relied entirely on broad ICD diagnostic codes extracted from administrative databases as proxy markers for mental health outcomes. These studies included De Giorgi et al., 2024; Hurtado et al., 2024; Kuzucan et al., 2026; Shapiro et al., 2025; Stanislaus et al., 2026; Tang et al., 2024; Ueda et al., 2024; and Xie et al., 2025 [19,20,22-24,26-28]. This structural constraint means that subtle day-to-day mood improvements, reductions in anxiety severity, and overall quality of life gains remain largely invisible across the majority of the included evidence. A psychiatric improvement that never triggered a formal diagnostic code change would simply not appear in these datasets, regardless of how clinically meaningful it may be to the patient.

Witaszek et al., 2024 [30] provided the most direct and clinically granular evidence in this review by actively measuring depression and anxiety, using the validated PHQ-9 and GAD-7 questionnaires [12,13] among 1,105 adult women undergoing anti-obesity pharmacotherapy in Poland. This study demonstrated that semaglutide users had significantly lower depression and anxiety scores than the rest of the study sample (10.84 ± 6.33 for depression and 9.80 ± 6.07 for anxiety). This scale-based signal is unique and valuable because it captures how patients actually feel rather than whether they received a new diagnostic label. Kuckuck et al., 2026, though outside our formal eligibility criteria due to its single-arm design, similarly reported improved mood and psychological well-being with liraglutide using validated scales, reinforcing this scale-based signal [35].

Longitudinal diagnostic stability across large registries provides secondary confirmation where scale data is absent. Taipale et al., 2026, provided the largest and most psychiatric-specific cohort in this review [25]. They analyzed 95,490 Swedish patients who all had pre-existing documented psychiatric diagnoses of depression, anxiety, or both at baseline [25]. They found that initiating semaglutide was associated with significantly reduced risk of psychiatric worsening over time, and that liraglutide showed a similar but less pronounced protective pattern. The stabilizing effect observed in patients who already had an established psychiatric diagnosis at baseline makes this finding clinically meaningful.

Objective Evidence on Psychiatric Hospitalization

Whenever we evaluate a true clinical impact of any drug on mental health, the most objective and meaningful endpoint is whether a patient requires inpatient psychiatric hospital admission. In contrast to the outpatient diagnostic codes, which are easily skewed by subjective diagnoses, varying billing practices, and surveillance bias, an inpatient psychiatric admission provides a hard, objective endpoint.

Only one study, Taipale et al., 2026, in this review, examined inpatient events directly [25]. Their linked national Swedish registry data showed that among 95,490 patients with fully established psychiatric histories, semaglutide use was associated with a meaningful reduction in hospitalization for a psychiatric cause or self-harm. Note that this outcome combined psychiatric and self-harm hospitalizations and did not isolate psychiatric admissions alone. GLP-1RA use also reduced the risk of self-harm specifically, though this secondary outcome overlaps the inpatient composite rather than adding to it. These findings are among the most clinically relevant in this review, as they address the most severe end of the psychiatric spectrum. It is the point at which mental health deterioration becomes life-threatening and resource-intensive for healthcare systems.

All other cohort studies in this review relied on administrative diagnostic codes, which structurally cannot distinguish between a true inpatient psychiatric admission and a routine outpatient clinic visit. The study by Ueda et al., 2024, tracked outcomes through national patient registers across Sweden and Denmark and showed a significant protective profile for suicide death and nonfatal self-harm [27]. However, this study also could not fully isolate pure inpatient psychiatric stays as a standalone endpoint. Similarly, Shapiro et al., 2025, indicated strict safety neutrality against both DPP-4 inhibitor and SGLT-2 inhibitor controls, but again could not isolate true psychiatric inpatient admissions [23].

This means the full scope of GLP-1RA effects on psychiatric hospitalization rates across different populations and healthcare systems remains only partially answered by the current literature. These findings strongly suggest an association between GLP-1RA use, metabolic stabilization, and reduced acute psychiatric decompensation. But causation cannot be established from observational data alone. Future studies should specifically track hospital admissions and prioritize hospitalization rates as a primary endpoint.

Clinical Recommendations and Future Research

The comprehensive findings of this review offer a clear and evidence-based framework for clinical decision-making across two distinct patient populations. First, for T2DM patients who are suffering from stable depression, anxiety, or controlled mood disorders, there is no empirical or epidemiological justification for withholding GLP-1RA therapy on psychiatric grounds [19,20,22-24,26,27].

The evidence from most of the studies consistently and strongly supports safety in this population. In many cases, the evidence points toward genuine psychiatric benefits through metabolic stabilization. These medications appear unlikely to destabilize mental health in this population, and based on the findings of Taipale et al., 2026, they may actively support long-term psychiatric stability [25].

Second, a more structured and cautious approach is warranted for patients who are pursuing weight management in specialized obesity clinics, given the divergent signals identified by Kornelius et al., 2024 [21]. Before initiating any anti-obesity regimen, clinicians may consider baseline mental health screening using validated instruments, especially PHQ-9 for depression [12] and GAD-7 for anxiety [13], as utilized in the Witaszek et al., 2024, study [30].

Establishment of this documented baseline is important for two reasons: First, it allows clinicians to distinguish true treatment-emergent psychiatric changes from pre-existing vulnerabilities that were simply not previously documented or formally diagnosed. Second, it creates a trackable reference point against which any subsequent mood changes can be objectively measured rather than relying on subjective patient or clinician impression.

Future clinical research should move beyond establishing safety and focus on precisely characterizing mood benefits and how best to measure them, as suggested by Taipale et al., 2026, and Witaszek et al., 2024 [25,30]. Dedicated prospective studies using validated, continuous mood rating instruments rather than administrative diagnostic codes are urgently needed to achieve this. 

Limitations

This review has several important structural limitations that must be explicitly acknowledged. First, all 11 primary studies included in this synthesis were observational studies. There was no randomized controlled trial included in the final studies with psychiatric safety or antidepressant efficacy as a primary endpoint. This means that while meaningful associations between GLP-1RA use and psychiatric outcomes can be identified, causation cannot be established. The observational nature of the evidence base is an inherent constraint that limits the strength of clinical inference that can be drawn from even the most robust registry findings.

Second, methodological heterogeneity existed across the 11 included studies. Follow-up duration varied markedly from about six months to a 5.2-year mean follow-up. Patient populations differed significantly, ranging from elderly U.S. Medicare beneficiaries aged 66 and older to young adult women in Poland. Comparator groups were inconsistent across studies, with some using SGLT-2 inhibitors, others using DPP-4 inhibitors, and others using standard-of-care or no-treatment controls. Definitions of what constituted a psychiatric endpoint also varied widely, making direct cross-study comparison methodologically challenging. This heterogeneity also raises the possibility of errors when synthesizing findings across populations and outcome definitions that are not directly comparable.

Third, reliance on administrative diagnostic codes and only partial hospitalization data limit the precision of both antidepressant-benefit and hospitalization findings, as discussed in the preceding Discussion section.

Fourth, this review was not prospectively registered (e.g., with PROSPERO), which limits transparency regarding pre-specified outcomes and methods relative to registered reviews. This review also did not apply a formal GRADE certainty-of-evidence framework, nor did it conduct formal testing for reporting bias. Funnel-plot methods for assessing reporting bias require a larger set of studies with comparable effect sizes than the 11 heterogeneous studies included here can provide. Additionally, our search was limited to three databases; other sources may have identified additional eligible studies. The exclusion of seven studies after quality appraisal, while based on prespecified design and scope criteria, also introduces the possibility of selection bias in the evidence contributing to our synthesis.

Finally, we must acknowledge potential geographic and database selection bias by focusing on the evidence from North America, the United Kingdom, Scandinavia, and Poland. Smaller localized or non-English language datasets may have been inadvertently excluded during the search and screening process. This geographic concentration may limit the generalizability of these findings to other populations, healthcare systems, and socioeconomic contexts where metabolic and psychiatric comorbidity patterns, cultural attitudes toward mental health help-seeking, and healthcare access may differ meaningfully from the settings represented in this review.

## Conclusions

The evidence synthesized in this review indicates that GLP-1 receptor agonists do not possess a class-wide neuropsychiatric risk profile. These agents consistently demonstrate either safety neutrality or a protective association against severe psychiatric decompensation and self-harm, tracked through global healthcare registries across a large multinational patient population. Isolated reports of increased psychiatric diagnostic codes appeared in one specialized obesity-focused cohort study. These elevations may partly reflect surveillance bias, stemming from the proactive mental health screening inherent in weight-management programs, though this was not directly tested, rather than an intrinsic pharmacological mechanism. Regarding psychiatric hospitalization, the question remains only partially answered, as most cohort studies relied on administrative diagnostic codes that could not cleanly isolate true inpatient psychiatric stays from outpatient reporting.

Regarding antidepressant potential, semaglutide was associated with lower depressive and anxiety symptom scores on validated psychometric tools. It also showed a significantly reduced risk of progression of psychiatric illness over time in patients with pre-existing mood disorders. However, the precise magnitude of benefit remains incompletely quantified, representing the highest priority gap for future research. Overall, GLP-1 receptor agonists appear to maintain a strong neuropsychiatric safety profile, with an emerging signal of antidepressant potential that remains limited to a single scale-based study among those formally included in this synthesis. This warrants cautious integration into future psychiatric and metabolic comorbidity management guidelines as further scale-based evidence emerges.

## References

[1] Kushner RF, Fink-Jensen A, Frenkel O, McGowan B, Goldman B, Overvad M, Wadden T (2024). Efficacy and safety of semaglutide 2.4 mg according to antidepressant use at baseline: a post hoc subgroup analysis. Obesity (Silver Spring).

[2] McElroy SL, Guerdjikova AI, Blom TJ, Mori N, Romo-Nava F (2024). Liraglutide in obese or overweight individuals with stable bipolar disorder. J Clin Psychopharmacol.

[3] Wadden TA, Brown GK, Egebjerg C (2024). Psychiatric safety of semaglutide for weight management in people without known major psychopathology: post hoc analysis of the STEP 1, 2, 3, and 5 trials. JAMA Intern Med.

[4] Fang W, Li Z, Xu J, Zhao J, Zhu H, Wang H (2025). Effect of liraglutide on depressive symptoms in overweight or obese patients with type 2 diabetes: protocol for a pilot randomized controlled trial. Front Endocrinol (Lausanne).

[5] Wadden TA, Oquendo MA, Kushner RF, Cao D, Karanikas CA, Kechter A, Murphy MA (2026). Psychiatric safety of tirzepatide in people with obesity and no known major psychopathology: a post hoc analysis of SURMOUNT. Obesity (Silver Spring).

[6] López-Ojeda W, Hurley RA (2024). Glucagon-like peptide 1: an introduction and possible implications for neuropsychiatry. J Neuropsychiatry Clin Neurosci.

[7] Badulescu S, Gill H, Shah H (2026). Semaglutide for the treatment of cognitive dysfunction in major depressive disorder: a randomized clinical trial. Med.

[8] McIntyre RS, Mansur RB, Rosenblat JD, Kwan AT (2024). The association between glucagon-like peptide-1 receptor agonists (GLP-1 RAs) and suicidality: reports to the Food and Drug Administration Adverse Event Reporting System (FAERS). Expert Opin Drug Saf.

[9] McIntyre RS, Mansur RB, Rosenblat JD (2025). Glucagon-like peptide-1 receptor agonists (GLP-1 RAs) and suicidality: a replication study using reports to the World Health Organization pharmacovigilance database (VigiBase®). J Affect Disord.

[10] (2026). FDA requests removal of suicidal behavior and ideation warning from glucagon-like peptide-1 receptor agonist (GLP-1 RA) medications. https://www.fda.gov/drugs/drug-safety-communications/fda-requests-removal-suicidal-behavior-and-ideation-warning-glucagon-peptide-1-receptor-agonist-glp.

[11] Page MJ, McKenzie JE, Bossuyt PM (2021). The PRISMA 2020 statement: an updated guideline for reporting systematic reviews. BMJ.

[12] Kroenke K, Spitzer RL, Williams JB (2001). The PHQ-9: validity of a brief depression severity measure. J Gen Intern Med.

[13] Spitzer RL, Kroenke K, Williams JBW, Löwe B (2006). A brief measure for assessing generalized anxiety disorder: the GAD-7. Arch Intern Med.

[14] World Health Organization (2004). ICD-10: International Statistical Classification of Diseases and Related Health Problems, 10th Revision, 2nd ed. ICD-10: International Statistical Classification of Diseases and Related Health Problems, 10th Revision, 2nd ed.

[15] World Health Organization (1978). International Classification of Diseases, Ninth Revision, Basic Tabulation List with Alphabetic Index. International Classification of Diseases, Ninth Revision, Basic Tabulation List with Alphabetic Index.

[16] Wells GA, Shea B, O'Connell D, Peterson J, Welch V, Losos M, Tugwell P (2000). The Newcastle-Ottawa Scale (NOS) for Assessing the Quality of Nonrandomised Studies in Meta-Analyses. https://ohri.ca/en/who-we-are/core-facilities-and-platforms/ottawa-methods-centre/newcastle-ottawa-scale.

[17] Sterne JA, Savović J, Page MJ (2019). RoB 2: a revised tool for assessing risk of bias in randomised trials. BMJ.

[18] Moola S, Munn Z, Tufanaru C (2024). Systematic reviews of etiology and risk. JBI Manual for Evidence Synthesis.

[19] De Giorgi R, Koychev I, Adler AI, Cowen PJ, Harmer CJ, Harrison PJ, Taquet M (2024). 12-month neurological and psychiatric outcomes of semaglutide use for type 2 diabetes: a propensity-score matched cohort study. EClinicalMedicine.

[20] Hurtado I, Robles C, Peiró S, García-Sempere A, Sanfélix-Gimeno G (2024). Association of glucagon-like peptide-1 receptor agonists with suicidal ideation and self-injury in individuals with diabetes and obesity: a propensity-weighted, population-based cohort study. Diabetologia.

[21] Kornelius E, Huang JY, Lo SC, Huang CN, Yang YS (2024). The risk of depression, anxiety, and suicidal behavior in patients with obesity on glucagon like peptide-1 receptor agonist therapy. Sci Rep.

[22] Kuzucan A, Tran V, Jones JT (2026). Association of GLP-1 receptor agonists with intentional self-harm in patients with type 2 diabetes: a Sentinel Distributed Database study. BMJ Open Diabetes Res Care.

[23] Shapiro SB, Yin H, Yu OH, Rej S, Suissa S, Azoulay L (2025). Glucagon-like peptide-1 receptor agonists and risk of suicidality among patients with type 2 diabetes: active comparator, new user cohort study. BMJ.

[24] Stanislaus C, Winther-Jensen M, Stanislaus S, Osler M, Coello K, Vinberg M (2026). GLP-1 receptor agonists and risk of suicide or suicide attempts - A nationwide cohort and self-controlled case series study. Mol Psychiatry.

[25] Taipale H, Taylor M, Lähteenvuo M, Mittendorfer-Rutz E, Tanskanen A, Tiihonen J (2026). Association between GLP-1 receptor agonist use and worsening mental illness in people with depression and anxiety in Sweden: a national cohort study. Lancet Psychiatry.

[26] Tang H, Lu Y, Donahoo WT (2024). Glucagon-like peptide-1 receptor agonists and risk for suicidal ideation and behaviors in U.S. older adults with type 2 diabetes : a target trial emulation study. Ann Intern Med.

[27] Ueda P, Söderling J, Wintzell V (2024). GLP-1 receptor agonist use and risk of suicide death. JAMA Intern Med.

[28] Xie Y, Choi T, Al-Aly Z (2025). Mapping the effectiveness and risks of GLP-1 receptor agonists. Nat Med.

[29] Battini V, Van Manen RP, Gringeri M (2023). The potential antidepressant effect of antidiabetic agents: new insights from a pharmacovigilance study based on data from the reporting system databases FAERS and VigiBase. Front Pharmacol.

[30] Witaszek T, Kłoda K, Mastalerz-Migas A, Babicki M (2024). Association between symptoms of depression and generalised anxiety disorder evaluated through PHQ-9 and GAD-7 and anti-obesity treatment in Polish adult women. Nutrients.

[31] Kim TH, Lee K, Park S (2024). Association between glucagon-like peptide-1 receptor agonists and risk of suicidality: a comprehensive analysis of the global pharmacovigilance database. Diabetes Obes Metab.

[32] Pierret AC, Mizuno Y, Saunders P (2025). Glucagon-like peptide 1 receptor agonists and mental health: a systematic review and meta-analysis. JAMA Psychiatry.

[33] Wang W, Volkow ND, Berger NA, Davis PB, Kaelber DC, Xu R (2024). Association of semaglutide with risk of suicidal ideation in a real-world cohort. Nat Med.

[34] Hooker SA, Neugebauer RS, Schmittdiel JA (2026). Comparative safety of glucose-lowering medications on depression in adults with type 2 diabetes. Diabetes Obes Metab.

[35] Kuckuck S, van Gerwen N, Oosterman JE (2026). Mental health changes after 4 months of weight loss treatment with the glucagon-like peptide-1 analogue liraglutide 3.0 mg. Diabetes Obes Metab.

